# Environmentally triggered genomic plasticity and capsular polysaccharide formation are involved in increased ethanol and acetic acid tolerance in *Kozakia baliensis* NBRC 16680

**DOI:** 10.1186/s12866-017-1070-y

**Published:** 2017-08-10

**Authors:** Julia U. Brandt, Friederike-Leonie Born, Frank Jakob, Rudi F. Vogel

**Affiliations:** Technische Universität München, Lehrstuhl für Technische Mikrobiologie, Gregor-Mendel-Straße 4, 85354 Freising, Germany

**Keywords:** *Kozakia baliensis*, Heteropolysaccharides, Pellicle, Ethanol/acetic acid tolerance, Adaptive evolution

## Abstract

**Background:**

*Kozakia baliensis* NBRC 16680 secretes a *gum-*cluster derived heteropolysaccharide and forms a surface pellicle composed of polysaccharides during static cultivation. Furthermore, this strain exhibits two colony types on agar plates; smooth wild-type (S) and rough mutant colonies (R). This switch is caused by a spontaneous transposon insertion into the *gumD* gene of the *gum*-cluster, resulting in a heteropolysaccharide secretion deficient, rough phenotype. To elucidate, whether this is a directed switch triggered by environmental factors, we checked the number of R and S colonies under different growth conditions including ethanol and acetic acid supplementation. Furthermore, we investigated the tolerance of R and S strains against ethanol and acetic acid in shaking and static growth experiments. To get new insights into the composition and function of the pellicle polysaccharide, the *polE* gene of the R strain was additionally deleted, as it was reported to be involved in pellicle formation in other acetic acid bacteria.

**Results:**

The number of R colonies was significantly increased upon growth on acetic acid and especially ethanol. The morphological change from *K. baliensis* NBRC 16680 S to R strain was accompanied by changes in the sugar contents of the produced pellicle EPS. The R:Δ*polE* mutant strain was not able to form a regular pellicle anymore, but secreted an EPS into the medium, which exhibited a similar sugar monomer composition as the pellicle polysaccharide isolated from the R strain. The R strain had a markedly increased tolerance towards acetic acid and ethanol compared to the other NBRC 16680 strains (S, R:Δ*polE*). A relatively high intrinsic acetic acid tolerance was also observable for *K. baliensis* DSM 14400^T^, which might indicate diverse adaptation mechanisms of different *K. baliensis* strains in altering natural habitats.

**Conclusion:**

The results suggest that the genetically triggered R phenotype formation is directly related to increased acetic acid and ethanol tolerance. The *polE* gene turned out to be involved in the formation of a cell-associated, capsular polysaccharide, which seems to be essential for increased ethanol/acetic tolerance in contrast to the secreted *gum*-cluster derived heteropolysaccharide. The genetic and morphological switch could represent an adaptive evolutionary step during the development of *K. baliensis* NBRC 16680 in course of changing environmental conditions.

**Electronic supplementary material:**

The online version of this article (doi:10.1186/s12866-017-1070-y) contains supplementary material, which is available to authorized users.

## Background

Gram-negative bacteria produce extracellular hetero- (HePS) or homopolysaccharides (HoPS), which are attached to the bacterial cell as capsular polysaccharide (CPS) or secreted into the environment as extracellular polysaccharide (EPS). Bacterial polysaccharides are important for the survival of bacteria, for instance in bacteria–host interaction, biofilm formation [1] and stress adaptation [2].

Acetic acid bacteria (AAB) are obligate aerobes and belong to the class of α-Proteobacteria. They are oxidative bacteria that strongly oxidize ethanol to acetic acid. AAB are well known for their ability to produce large amounts of EPSs, either HoPS, like dextrans, levans [3–5] and cellulose, or different kinds of HePS, such as acetan [6] and gluconacetan [7]. Furthermore, a variety of AAB has the ability to grow floating on the surface of a static culture by producing a pellicle enabling a high aeration state. The pellicle consists of an accumulation of cells, which are tightly associated with each other by capsular polysaccharides as connecting element. The pellicle CPS can be a HoPS of cellulose, which is produced by many *Komagataeibacter* species, like *Komagataeibacter (K.) xylinus* [8] (formerly *Gluconobacter (G.) xylinum* [9]), or a HePS, such as produced by many *Acetobacter* strains [10–12]. This HePS can be composed of different sugar monomers, like the HePS of *A. tropicalis* SKU1100. which consists of glucose, galactose, and rhamnose [13] or of *A. aceti* IFO3284 that contains only glucose and rhamnose [10].

The genes involved in the cellulose pellicle biosynthesis are arranged in an operon structure, like the acs operon [13] or the bcs operon [14], and widely studied. It is assumed that the genes involved in the synthesis of other pellicle HePS in AAB are assigned to a particular cluster, called *pol*-cluster. Deeraksa et al. (2005) could show, that the pellicle HePS produced by *A. tropicalis* SKU1100 could be traced back to a gene cluster, *polABCDE*, which is required for pellicle formation. In this operon, the *polABCD* genes showed high similarity to *rfbBACD* genes, which are involved in dTDP-l-rhamnose biosynthesis. The downstream located *polE* gene showed only low similarity to known glycosyltransferases, whereas a transposon-induced disruption of the *polE* gene resulted in a non pellicle forming strain, due to the absence of CPS production. Instead of the CPS, however, the Pel^−^ strain showed a smooth-surfaced colony and the HePS was now secreted into the medium, which had the same composition as the capsular pellicle polysaccharide [11].

*Acetobacte*r species are further known to exhibit high natural mutation frequencies [15, 16], also resulting in the formation of two or more different colony types. *A. pasteurianus* IFO3284 produces two altering types of colonies on agar medium that are inter-convertible by spontaneous mutation; a rough surface colony that can produce a pellicle (R strain) and smooth surface colony, which cannot produce a pellicle (S strain) [17]. Furthermore the R strains tolerate higher concentrations of acetic acid, whereas the pellicle formation is directly related to acetic acid resistance [18, 19]. It is further assumed that the pellicle CPS functions as a barrier-like biofilm against passive diffusion of acetic acid into the cells [18].

Microorganisms constantly face many difficult challenges, due to changing environmental conditions. The capacity to maintain functional homeostasis is essential for their survival. Recently, we have shown that the AAB *Kozakia (K.) baliensis* NBRC 16680 forms large amounts of a soluble unique HePS in the medium, as well as a pellicle during static cultivation [20]. Furthermore, *K. baliensis* NBRC 16680 forms a second type of colony form: a non-HePS producing rough-surfaced colony (R strain), caused by a transposon insertion into the *gumD* gene of the corresponding *gum*-like HePS cluster. The reason for this transposon insertion is unclear, whereby it was assumed that the transposon insertion represents a directed event, triggered by external factors.

Therefore, we investigated in this study, whether the morphology switch of *K. baliensis* NBRC 16680 is a random event, or triggered by environmental adaptations. In order to check if the morphological switch is connected to the formation of a CPS used in pellicle formation, we performed a *ΔpolE* deletion in *K. baliensis* NBRC 16680 R via a two step marker less gene deletion system. The different mutants were investigated regarding their growth and EPS production under different growth conditions including ethanol and acetic acid stress.

## Methods

### Bacterial strains, culture media, and culture conditions.

*K. baliensis* (NBRC 16680; National Institute of Technology and Evaluation (NITE) Biological Resource Center, Japan, DSM 14400; German Collection of Microorganisms and Cell Cultures(DSMZ)), as well as a mutant strain of *K. baliensis* NBRC 16680 R (*ΔgumD*) [20], with a rough phenotype, were used in this study. *K. baliensis* and its derivatives were grown at 30 °C in NaG media consisting of 20 g/L sodium gluconate, 3 g/L yeast extract, 2 g/L peptone, 3 g/L glycerol, 10 g/L mannitol and a pH adjusted to 6.0. *E. coli* strain TOP10 (Invitrogen, Karlsruhe, Germany), was grown at 37 °C and 180 rpm on a rotary shaker in LB medium consisting of 5 g yeast extract, 5 g NaCl and 10 g peptone. For selection of plasmids in *E. coli* TOP10. 50 μg/mL kanamycin was added to the LB medium. For selection of recombinant *K. baliensis* strains 50 μg/mL kanamycin or 60 μg/mL Fluorocytosin (FC) were used.

### Convertibility of *K. balie*nsis NBRC 16680 from wild-type (S) to rough strains (R)

The number of R strains was determined during/after cultivation of *K. bali*ensis NBRC 16680 in standard NaG medium, NaG medium supplemented with 3% ethanol (NaG-EtOH) or 0.4% acetic acid (NaG-AA), respectively. *K. baliensis* NBRC 16680 was first cultivated overnight in 10 ml of unmodified NaG medium at 30 °C (200 rpm). About 1 × 10^8^ CFU/mL seed culture was afterwards transferred to 10 ml of standard or modified NaG medium, respectively. The flasks were incubated at 30 °C with rotary shaking at 200 rpm for 48 h and samples adducted at 0, 24 and 48 h. Cell numbers of wild-type and rough colonies (*ΔgumD*) were counted on non-modified NaG agar plates. Each growth experiment in liquid culture was performed thrice in separate assays, while each assay contained further three technical plating replicates.

For targeting the transposon insertion side, random colony PCRs of 23 rough colonies were carried out with Phire Hot start DNA polymerase (Thermo Fisher scientific; Waltham, USA). A primer set of a genomic primer (G4F_Fw) and a primer, targeting the mobile element (TE_Rv) were used; primers are listed in Additional file 1. PCR products were subsequently sequenced via Sanger sequencing by GATC Biotech (Konstanz, Germany).

### Deletion of the *polE* gene with a two step marker less gene deletion system

For plasmids preparation, the GeneJET Plasmid Miniprep Kit (Thermo Fisher scientific, Waltham, USA) was used. Genomic DNA from *K. baliensis* NBRC 16680 R was extracted with the E.Z.N.A. Bacterial DNA Kit (Omega Biotek, Norcross, USA) and DNA purification was done with the E.Z.N.A. Cycle-Pure Kit (Omega Bio-tek, Norcross, USA). Restriction enzymes, DNA ligase, and alkaline phosphatase (FastAP) were obtained from Fermentas (Waltham, USA). PCRs were performed according to the Phusion High-Fidelity DNA Polymerase manuals from New England Biolabs (Frankfurt, Germany). For construction of the deletion vector, a fusion PCR technique was used to ligate the PCR products of flanking regions according to a long flanking homology (LFH) protocol [21, 22]. The length of the homology sequences were 20 bp. Primers are listed in Additional file 1. An enzyme-free cloning technique [23] was used for the further construction of the deletion vector, with the pKOS6b plasmid as basis, including a multiple cloning site (MCS), a kanamycine resistance gene (KM^R^) and the *codA* and *codB* gene (Additional file 2A) [24]. Flanking regions of the *polE* gene, covering approximately 950 bp of the upstream and downstream region of the *polE* gene were amplified via PCR. The upstream region of 957 bp was amplified with primer P1_*polE*_KpnI_Fw and P2-*polE*_Rv, and a second primer set amplified the downstream region (968 bp) of the *polE* gene, containing the primers P3_*polE*_Fw and P4-*polE*_XbaI_Rv (Additional file 1 & Additional file 2A). In the following step, a LFH PCR [21] was performed with P1_*polE*_KpnI_Fw and P4-*polE*_XbaI_Rv to merge the two previously amplified fragments (1898 bp). The fused fragment, as well as the pKOS6b vector, were both digested (KpnI, XbaI) and finally ligated. The resulting deletion vector, pKOS6bΔ*polE*, was verified via sanger sequencing (pK18MCS_Fw & pK18MCS_Rv), and further amplified in *E. coli* TOP 10. The transformation of pKOS6bΔ*polE* into *K. baliensis* NBRC 16680 R, was carried out by electroporation [25–28] with Gene Pulser Xcell™ Electroporation Systems from Bio Rad (München, Germany). Therefore, cells were inoculated to an OD_600_ of 0.3 in NaG medium and finally grown to an OD_600_ of 0.9. The culture was centrifuged at 5000 g, at 4 °C for 10 min, and washed three times in 1 mM HEPES buffer (pH 7). Cells were resuspended in 1 mM HEPES buffer, supplemented with ¼ volume of glycerin and shock frozen in 50 μL aliquots. The electroporation took place in cuvettes with 2 mm electrode distance from Bio Rad (München, Germany). The electroporation was carried out under constant conditions: 2.5 kV, 25 μF, and 400 Ω. Fresh enriched NaG medium (450 mM mannitol, 15 g/L yeast extract, 15 mM CaCl_2_, 10 mM MgSO_4_ and 6 mM glycerin) was added immediately after the pulse. The treated cells were incubated on a rotary shaker over 14 h and subsequently plated on NaG plates containing 50 μg/mL kanamycin for the first selection step with an incubation time of 48 h. During the first recombination step on NaG-Kan plates, a random chromosomal integration of the plasmid took place, which was checked by colony PCR using a specific primer set of a plasmid (pK18MCS_Fw or pK18MCS_Rv) and a genome (CL_*polE*_Fw or CL_*polE*_Rv) specific primer. Phire Hot start DNA polymerase (Thermo Fisher scientific; Waltham, USA) was used for colony PCR reactions, to screen for mutants or to confirm integration of the deletion vector into the genome. PCR products were sequenced via sanger sequencing by GATC Biotech (Konstanz, Germany). The positive clones were further grown on NaG-plates with 60 μg/ml FC, to drive the directed loss of the plasmid and the final selection of *K. baliensis* NBRC 16680 R Δ*polE* mutant colonies. After 3 days of incubation, the correct Δ*polE* mutant colonies could be identified via colony PCR, with a genome specific primer set (CL_*polE*_Fw & CL_*polE*_Rv) resulting in a 1950 bp fragment for the Δ*polE* mutant and a 3000 bp fragment for *K. baliensis* NBRC 16680 R (Additional file 2C).

### Growth behavior of different *K. baliensis* strains in acetic acid and ethanol

*K. bali*ensis DSM 14400. NBRC 16680. the *ΔgumD* mutant [20] and the *ΔpolE* mutant strain (see 2.3) were grown on NaG agar plates, directly plated from the particular cryo stock, with either ethanol or acetic acid, in different concentrations. The ethanol supplemented plates contained 1% - 10% of ethanol (*v*/v, at intervals of 1%) and the acetic acid supplemented plates 0.1% - 1% of acetic acid (*v*/v, at intervals of 0.1%). Each strain was streaked onto the plates from cryo-stocks and incubated for 3 days at 30 °C.

Furthermore, a static cultivation in NaG medium with 3% ethanol and 0.6% acetic acid was carried out. The *K. baliensis* NBRC 16680 strain, R mutant [20] and the *ΔpolE* mutant (see below) were grown as seed cultures in unmodified NaG media, overnight. Cultures were inoculated with an OD_600_ 0.3 and cultivated up to 0.9, respectively. For static cultures, 300 μl of the seed culture were inoculated into 3 ml NaG medium and cultivated statically at 30 °C. Cells were harvested by centrifugation at 6000 g and cell pellets were dried overnight at 120 °C. The dry weight was measured each day, over a time span of 7 days.

### Analysis of HePS composition

Main cultures of *K. baliensis* NBRC 16680. *ΔgumD* mutant [20] and *ΔpolE* mutant (see below) were performed in 500 mL Erlenmeyer flasks with 50 mL of modified NaG media, inoculated with 500 μl of the pre-cultures and kept at 30 °C in a rotary shaker (200 rpm) for 32 h. Afterwards, cells were removed and the EPS containing supernatants were precipitated with cold ethanol (2:1, *v*/v) and kept overnight at 4 °C. This step was repeated three times, followed by a dialysis step (MWCO 14 kDa) of the recovered (centrifugation) and in ddH_2_O re-dissolved HePS. Finally, the purified HePSs were lyophilized and quantified by weighing. To obtain large amounts of pellicle EPS*, K. baliensis* NBRC 16680 R [20] and the *ΔpolE* mutant (see 2.3) were cultured in unmodified NaG medium in cell culture flasks (Greiner Bio-One, Austria), to ensure a large surface for oxygen supply. Briefly, 10% of the seed culture was inoculated to 30 mL NaG medium and incubated statically at 30 °C for 14 days. The pellicle EPS was purified from the culture and cells were separated from EPS via ultra-sonification (10 min) and mechanical disruption, related to the method of IAI Ali, Y Akakabe, S Moonmangmee, A Deeraksa, M Matsutani, T Yakushi, M Yamada and K Matsushita [11]. The culture was centrifuged (10 min, 10.000 g) and the supernatant was saved in another flask. The cell pellet was washed 2 times with 10 mM HEPES buffer (pH 7), and suspended in the same buffer. The suspension was again ultra-sonicated for 10 min, followed by a centrifugation step for 10 min, 13.000 g. The resulting supernatant was combined, with the present supernatant from the first centrifugation, and EPS was precipitated with cold ethanol (2:1, *v*/v) and kept overnight at 4 °C. This step was repeated three times, followed by a dialysis step (MWCO 14 kDa) of the recovered (centrifugation) and in ddH_2_O re-dissolved EPS.

The monosaccharide composition of the isolated *K. baliensis* NBRC 16680 R pellicle or secreted HePS (NBRC 16680. *ΔpolE*) was investigated via high performance liquid chromatography (HPLC). For HPLC analysis the purified polysaccharide samples were hydrolyzed with 10% of perchloric acid over 7 h at 100 °C, followed by a centrifugation step (4 °C, 10 min, 13,000 g) for removal of possible impurities, such as proteins. For the HPAEC analysis polysaccharide samples were hydrolyzed with 10% of perchloric acid over 2 h at 100 °C, as well followed by a centrifugation step (4 °C, 10 min, 13,000 g). The samples were further dissolved (1:10 or 1:100). The supernatant was analyzed using a Rezex RPM column (Phenomenex, Germany) coupled to a refractive index (RI) detector (Gynkotek, Germany) corresponding to the method of [29]. Sugar monomers were identified according to their retention time using suitable monosaccharide standards (D-glucose, D-galactose, D-mannose, D-rhamnose). The mobile phase was water, with a flow rate of 0.6 mL/min.

## Results

### Mutation of *K. baliensis* NBRC 16680 from S to R phenotype in dependence of different growth conditions

**Fig. 1:**
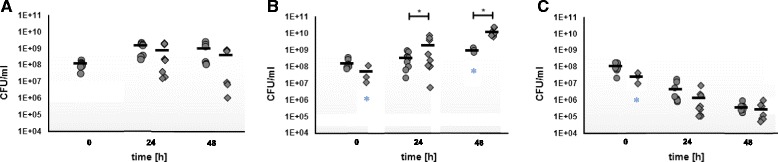
Growth of *K. baliensis* NBRC 16680 in different media and influence of the provided carbon source on the morphology switch to a rough colony morphology (*ΔgumD*). **a** Growth of *K. baliensis* NBRC 16680 in NaG medium, *n* = 9 replica: wild-type (●), *ΔgumD* (♦), mean value (▬). **b** Growth of *K. baliensis* NBRC 16680 in NaG medium with ethanol (NaG-EtOH, 3%), *n* = 9 replica, * means that only *n* = 3 replica were above the detection limit of 10^4^ CFU/mL: wild-type (●), *ΔgumD* (♦), mean value (▬).Asterisks centered over the error bars indicate the relative level of the *p*-value. In general, “*” means *p* < 0.05. (**c**) Growth of *K. baliensis* NBRC 16680 in NaG medium with acetic acid (NaG-AA, 0.4%), *n* = 9 replica, * means that only *n* = 3 replica were above the detection limit of 10^4^ CFU/mL: wild-type (●), *ΔgumD* (♦), mean value (▬)

In *K. baliensis* NBRC 16680 spontaneous mutations occur, which cause a non-slimy phenotype, referred as rough (R) strain. In a previous work we have demonstrated, that this mutation can result from a transposon insertion in the *gumD* gene of the HePS forming cluster of *K. baliensis* NBRC 16680 [20]. The *gumD* gene encodes the first step of HePS formation, whereas a loss of the functional *gumD* gene leads to a total disruption of the HePS production and secretion in *K. baliensis* NBRC 16680. To clarify, if this mutation is a random event, or if it is a directed mutation possibly triggered by environmental factors, we performed an experimental series using different growth media. Different sugar combinations were tested (see 2.2) as well as stress inducing conditions like growth in ethanol (3%) or in acetic acid (0.6%) supplemented media. In addition, dilutions of the cryo-culture of *K. baliensis* NBRC 16680 were directly plated on NaG plates. A distinction was made between slimy glossy wild-type colonies (S) and rough dull mutant colonies (R), which were transparent, held against the light. The NaG plates with the directly plated dilutions of the cryo-cultures from *K. baliensis* NBRC 16680 showed only wild-type colonies. At 0 h, i. e. after inoculation with the overnight culture, solely wild-type colonies could be identified in standard NaG-medium, with cell numbers of about 1 × 10^8^ CFU/mL (Fig. 1a). On the contrary, after inoculation of *K. baliensis* NBRC 16680 in NaG medium supplemented with ethanol or acetic acid, rough colonies could be detected at time point 0 in one of the three biological replicates, respectively (Fig. 1b*c*), which could have resulted from a too long contact to (and concomitant mutations in response to) acetic acid or ethanol. At 24 h the cell numbers of rough and wild-type strains were around 1,4 × 10^9^ CFU/mL in the NaG medium and bacteria merged already into the starvation phase, with final cell counts of about 1 × 10^9^ CFU/mL after 48 h (Fig. 1a). In standard NaG medium the number of wild-type colonies exceled the number of mutant colonies or showed approximately equal numbers of wild-type and mutant colonies. During stress-inducing conditions (NaG-EtOH, NaG-AA), however, for NaG-EtOH an inverted picture emerged. At both survey marks (24 h, 48 h) significantly more mutant (R) than wild-type colonies were detectable, with around ten-power difference (Fig. 1b). After 48 h only in three of nine cases, wild-type colonies with a detection limit above of 10^4^ CFU/mL could be detected (Fig. 1b*). In the NaG-AA medium, a continuous reduction of the cell numbers could be observed, in which the number of wild-type colonies always exceeded the number of mutated colonies (Fig. 1c).

**Fig. 2:**
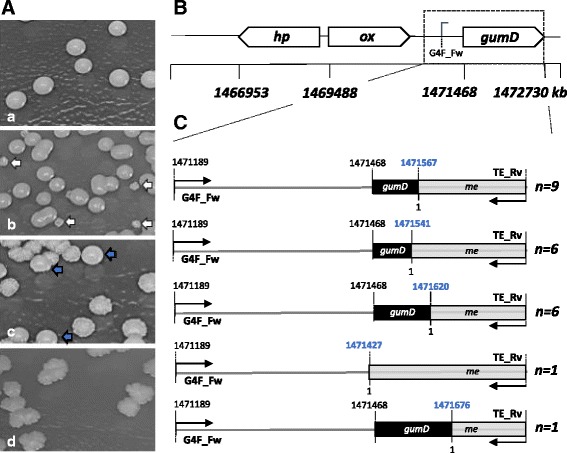
Morphology and genetic switch of *K. baliensis* NBRC 16680 wild-type during cultivation in different media (**a**) In (*a*), growth of *K. baliensis* NBRC 16680 on NaG agar plates, plated at time point one after inoculation in NaG medium (0 h). Growth of *K. baliensis* NBRC 16680 on NaG agar plates, plated after 48 h of incubation in NaG medium (*b*), NaG-AA medium (*c*) and NaG-EtOH medium (*d*). Rough mutant colonies (*ΔgumD*) are indicated via a *white arrow*. Wild-type colonies are marked with a *grey arrow*. **b**) Shows a section of the *gum*-cluster of *K. baliensis* NBRC 16680 with the genomic location of the *gumD* gene (1471468–1,472,730 bp), oxidoreductase gene (*ox*, 1,469,488–1,470,627 bp) and a gene coding for a hypothetical protein (*hp*, 1,467,916–1,469,289 bp), based on JU Brandt, Jakob, F., Behr, J., Geissler, A.J., Vogel, R.F. [20]. Random colony PCRs of the respective rough colonies were carried out, targeting the transposon insertion side, using a genomic primer (G4F_Fw) and a primer, targeting the mobile element (TE_Rv). PCR products were subsequently sequenced. (**c**) Shows a schematic representation of the transposon insertion at the *gumD* locus in the rough colonys of *K. baliensis* NBRC 16680. The mobile element (*me*) is shown as grey bar with the corresponding insertion locus written in bold *blue*. The frequency of the found insertion site is marked on the left side, as *n* = x

In order to verify a possible integration of a mobile element in the *gumD* gene [20], or in front of the *gumD* gene, random colony PCR reactions were carried out, with a forward primer (G4F_Fw) targeting a location in front of the *gumD* gene (1.471.189–1.471.208 bp) and a reverse primer (TE-Rv) targeting the mobile element (Additional file 1) (Fig. 2b). Subsequently, the obtained PCR fragments were sequenced. For each mutated colony, a transposon insertion in the region of the *gumD* gene could be identified (Fig. 2c). These insertions, however, were not always located at the same site, but in a defined region of about 300 bp, around and in the *gumD* gene (Fig. 2c). Furthermore, it could be observed that in certain areas an integration of the mobile element occurred more often than in other areas, like up to nine times at 1471567 bp.

### Influence of the *polE* gene on HePS formation and composition of *K. baliensis* NBRC 16680

**Fig. 3:**
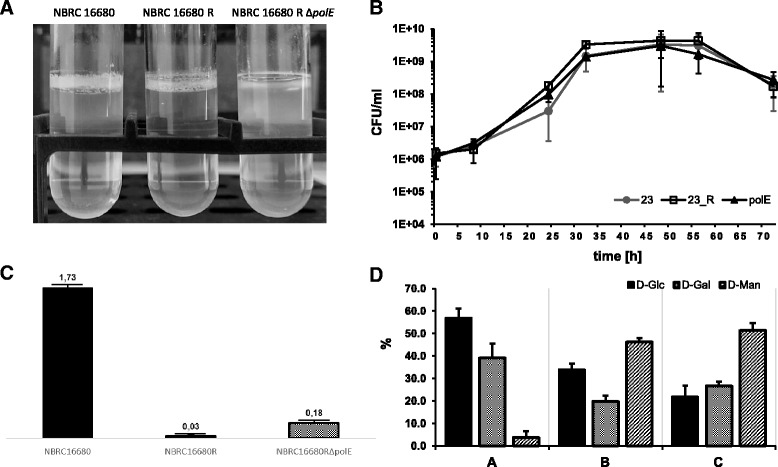
Comparison of *K. baliensis* NBRC 16680 wild-type, the rough mutant strain and the *polE* deficient strain. **a** Phenotyps of *K. baliensis* NBRC 16680 during static cultivation of wild-type, rough mutant (*K. baliensis* NBRC 16680 R) and rough *ΔpolE* mutant strain (*K. baliensis* NBRC 16680 R *ΔpolE*). **b** Growth behavior of the different *K. baliensis* strains (NBRC 16680 (•), NBRC 16680 R (□), NBRC 16680 R *ΔpolE*(▲)) in NaG medium. (**c**) Amount of precipitated HePS [g/L] from different *K. baliensis* strains (NBRC 16680. NBRC 16680 R, NBRC 16680 R *ΔpolE*) during growth in NaG medium. (**d**) Neutral sugar composition of the isolated HePS for different *K. baliensis* strains during shaking or static cultivation. The proportions of the monomer occurring in the particular HePS are represented as percentage. (*a*) NBRC 16680 (shaking), (*b*) NBRC 16680 R (static), (*c*) NBRC 16680 R *ΔpolE* (shaking)

In order to decode the role of the *polE* gene during pellicle formation of *K. baliensis* NBRC 16680 a *polE* deletion was carried out (see 2.3), with a markerless deletion system established by Kostner et al. [22, 24]. The pellicle forming ability of *K. baliensis* NBRC 16680 R Δ*polE* was further analyzed under static conditions in NaG-medium for 5 days. The growth behavior, as well as the HePS production, was compared with the wild-type and the rough mutant strain of *K. baliensis* NBRC 16680. All three *K. baliensis* strains showed pellicle formation after three days (Fig. 3a). For the wild-type strain of *K. baliensis* NBRC 16680 as well as for the rough mutant strain, a distinct pellicle formation at the edge of the test-tube was visible, which spreads over the entire boundary surface after three days. *K. baliensis* NBRC 16680 R Δ*polE* showed only a slight pellicle production (Fig. 3a). The physiology of the pellicle was different from the other two *K. baliensis* strains, instead of a surface spanning layer, only a loose conglomerate was present. The time for pellicle formation, also varied between the three strains. In comparison to the other *K. baliensis* strains, the pellicle of *K. baliensis* NBRC 16680 R Δ*polE* was only scarcely visible after three days of static incubation. During cultivation of the three *K. baliensis* strains (NBRC 16680, NBRC 16680 R and RΔ*polE*) under shaking conditions, no significant aberration in the growth behavior could be observed (Fig. 3b). In case of the HePS formation in shaking cultures with NaG medium, after 48 h a slight HePS production of *K. baliensis* NBRC 16680 R Δ*polE* could be demonstrated, with HePS amounts of 180 mg/L (Fig. 3c). The rough mutant *K. balien*sis strain, which differs only in the presence of an intact *polE* gene from *K. baliensis* NBRC 16680 R Δ*polE*, showed no EPS production in shaking cultures (Fig. 3c). The wild-type strain of *K. baliensis* NBRC 16680 with an intact *gum*- and *pol*-cluster, showed the highest EPS production under shaking conditions, with 1,73 g/L EPS.

*K. baliensis* NBRC 16680 HePS was furthermore isolated from shaking cultures and the monomer compositions were determined. It was possible to obtain pellicle EPS from the rough mutant strain (*K. baliensis* NBRC 16680 R), as well as EPS from shaking cultures from the *K. baliensis* NBRC 16680 wild-type strain and the *polE* deficient mutant, since for *K. baliensis* NBRC 16680 R, no EPS formation could be detected under shaking conditions. Because of the impaired pellicle formation of *K. baliensis* NBRC 16680 R:Δ*polE* (even during 14 days of cultivation in 30 mL cell culture flasks), no adequate amounts of EPS could be isolated for the monomer analysis. The monomer composition of wild-type *K. baliensis* NBRC 16680 HePS was composed of D-glucose, D-mannose and D-galactose (Fig. 3d). The statically cultivated rough mutant strain of *K. baliensis*, which is not able to produce HePS via the *gum*-cluster, showed a divergent monomer distribution, with generally higher amounts of D-mannose (46,2 ± 3,20%), and a consequently lower D-glucose (33,9 ± 5,03%) and D-galactose (19,9 ± 1,83%) level (Fig. 3d). The HePS derived from shaking cultures of the *polE* deficient *K. baliensis* NBRC 16680 R mutant showed a similar monomer distribution as the pellicle HePS of *K. baliensis* NBRC 16680 R, but with slightly variable percentages (Fig. 3d). Compared to *K. baliensis* NBRC 16680 R, a smaller proportion of D-glucose (21,8 ± 1,03%) was observable, while the proportion of D-mannose (51,4 ± 1,20%) was still higher.

### Physiological effects of Δ*gumD* and Δ*gumD* + Δ*polE* mutations

**Fig. 4:**
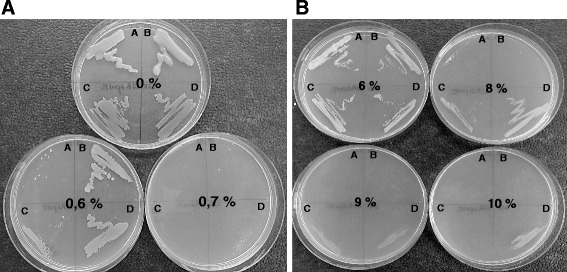
Effect of acetic acid and ethanol on the growth behavior of different *K. baliensis* strains (**a**) Effect of acetic acid on growth of different *K. baliensis* strains (DSM 14400 (*a*), NBRC 16680 (*b*), NBRC 16680 R *ΔpolE* (*c*), NBRC 16680 R (*d*)) at different acetic acid concentrations (0%, 0.6%, 0.7%). (**b**) Effect of ethanol on growth of different *K. baliensis* strains (DSM 14400 (*a*), NBRC 16680 (*b*), NBRC 16680 R *ΔpolE* (*c*), NBRC 16680 R (*d*)) at different ethanol concentrations (6%, 8%,9%, 10%)

Since the insertion of a mobile element into the *gumD* gene of the *gum*-cluster of *K. baliensis* NBRC 16680 appears to be a mechanism affected by external factors, the question arises, whether this is a physiological adaptation of the bacterium to changes in its external environment. Therefore, the growth behavior of the different *K. baliensis* strains, under different cultivation conditions, was investigated. *K. baliensis* NBRC 16680 and DSM 14400, *K. baliensis* NBRC 16680 R and the Δ*polE* mutant of the rough *K. baliensis* strain were plated on NaG-medium plates, which were supplemented with different acetic acid (0.1%, 0.2%, 0.3%, 0.4%, 0.5%, 0.6%, 0.7%, 0.8%, 0.9% and 1%) and ethanol (1%, 2%, 3%, 4%, 5%, 6%, 7%, 8%, 9% and 10%) concentrations. Furthermore, the growth behavior of *K. baliensis* DSM 14400 was additionally tested, to investigate the variability in the growth behavior between two different wild-type strains of *K. baliensis*. *K. baliensis* DSM 14400 forms a HePS which is also composed of D-glucose, D-galactose and D-mannose, but with a deviating ratio compared to NBRC 16680 [20]. After a cultivation period of three days, distinct differences in the growth behavior of the different strains could be observed. Acetic acid had a significant influence on the growth of the different *K. baliensis* NBRC 16680 strains, while 0.7% acetic acid was the highest concentration, at which colony formation on agar plates was still possible for *Kozakia* strains NBRC 16680 R and DSM 14400 (Fig. 4a). The *K. baliensis* NBRC 16680 wild-type and the Δ*polE* mutant of the rough *K. baliensis* NBRC 16680 strain were not able to form colonies above 0.6% of acetic acid. In case of growth on NaG-EtOH plates, *K. baliensis* NBRC 16680 R and *K. baliensis* NBRC 16680 Δ*polE*, showed the highest EtOH tolerance, whereas *K. baliensis* NBRC 16680 R could even grow at 10% EtOH. Both wild-type strains of *K. baliensis* (NBRC 16680, DSM 14400) showed only poor growth behavior upon 7% EtOH and no growth at all, above 8% EtOH in the medium (Fig. 4b).

**Fig. 5:**
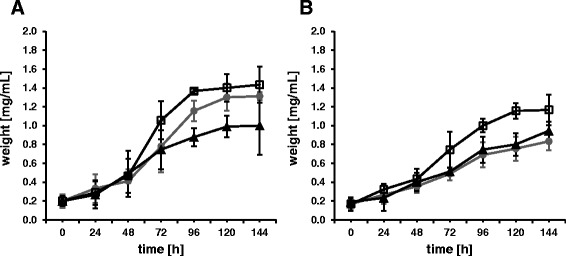
Growth behavior of different *K. baliensis* strains during static cultivation in altered media (**a**) Growth behavior of the different *K. baliensis* strains (NBRC 16680 (•), NBRC 16680 R (□), NBRC 16680 R *ΔpolE*(▲)) during static cultivation in NaG medium with acetic acid (0.6%). (**b**) Growth behavior of the different *K. baliensis* strains (NBRC 16680 (•), NBRC 16680 R (□), NBRC 16680 R *ΔpolE*(▲)) during static cultivation in NaG medium with ethanol (3%) The growth was monitored by measuring the dry weight of cells

Furthermore, the effect of acetic acid and ethanol on the growth behavior of the different *K. baliensis* strains was monitored under static cultivation, by measuring the dry weight of the cells. It has to be noted, that the secreted, *gum*-cluster based HePS of the wildtype strain from *K. baliensis* NBRC 16680 was largely removed by centrifugation in advance. However, it can still slightly contribute to the dry weight as a small residue, which is still connected with the bacteria. In the NaG medium with 0.6% of acetic acid, the rough mutant strain displayed the fastest growth and reached the highest dry weight, after 7 days (1,77 ± 0.19 mg/ml). The wild-type strain of *K. baliensis* NBRC16680 showed slightly reduced final dry weight (1,64 ± 0.04 mg/ml), but an offset log phase resulting in a slower growth rate than the rough mutant strain (Fig. 5a). Similar results were obtained for the growth of the corresponding strains in NaG medium with 3% ethanol. *K. baliensis* NBRC16680 R exhibited the fastest growth, with a final dry weight of 1,17 ± 0.16 mg/ml. For *K. baliensis* NBRC16680 and the Δ*polE* mutant strain, a markedly reduced growth could be demonstrated (Fig. 5b).

## Discussion

*K. baliensis* NBRC 16680 produces and secretes large amounts of HePS via a *gum*-cluster encoded HePS biosynthesis. By insertion of a mobile element into the *gumD* locus of *K. baliensis* NBRC 16680 a mutant strain of *K. baliensis* is formed (*K. baliensis* NBRC 16680 R), which is unable to produce and secrete the *gum*-cluster derived HePS [20]. After cultivation of *K. baliensis* NBRC 16680 in standard or modified media (NaG, NaG-EtOH, NaG-AA), *K. baliensis* NBRC 16680 rough mutant strains were more frequently found in the presence of ethanol in the medium. For AAB an induced loss of various physiological properties has been observed, such as acetic acid resistance [30], ethanol oxidation, pellicle formation [31], and bacterial cellulose synthesis. The genetic mechanisms behind these instabilities is often unclear. Takemura et al. [32] reported that the loss of the ethanol oxidation ability in *A. pasteurianus* NC11380 occurs by an insertion of a mobile element into the alcohol dehydrogenase-cytochrome c gene, resulting in the loss of alcohol dehydrogenase activity. This has also been demonstrated for *A. pasteurianus* NCI 1452, where an insertion sequence element (IS) is associated with the inactivation of the alcohol dehydrogenase gene [33]. Gene inactivation provoked by transposable elements could also be demonstrated for EPS forming clusters of AAB, like the cellulose synthase operon. In *A. xylinum* ATCC 23769, an IS element caused insertions 0.5 kb upstream of the cellulose synthase gene, associated with spontaneous cellulose deficiency [34]. In both cases, it was not possible to sustain revertants of the cellulose synthesis and ethanol oxidation insufficient mutant strains, possibly as a consequence of remaining directed repeats (DR) of the IS element after relocation. Also in case of one previously investigated *K. baliensis* NBRC 16680 R mutant strain, the formation of directed repeats at the transposon insertion side could be observed [21]. Moreover, insertions of the mobile element in front of the *gumD* locus of the R strains as detected in the present study could result in a blocked transcription of *gumD* due to the presence of an energy-rich stem loop structure in the mobile element possibly causing *rho*-independent transcription termination [20]. It can actually not be ruled out that further mutations in the genome of *K. baliensis* NBRC 16680 simultaneously occurred. However, transposon insertion at the *gumD* locus of *K. baliensis* NBRC 16680 seems to be directly involved in R phenotype formation, since all of the 23 PCR checked R mutants exhibited the transposon insertion side at the *gumD* locus.

Morphotype variations, including the transition from a mucoid to a non-mucoid phenotype, are common events within the family of *Acetobacteraceae*, especially for *Acetobacter* species. This phenotypic change is often connected with the ability of the bacteria to form a pellicle on the medium surface [10, 11]. The pellicle is an assemblage of cells that permits them to float on the medium surface during static cultivation and ensures a high state of aeration. It was also shown that pellicle production could be associated with a change from a smooth phenotype to the rough, pellicle-forming strain. This change is accompanied by a transformation from secreted EPS to CPS, which could serve as a better barrier against ethanol [18]. Also the rough mutant of *K. balien*sis NBRC 16680 is still able to form a pellicle under static cultivation [20], suggesting that a second cluster, instead of the *gum*-cluster, is responsible for pellicle construction. For different *Acetobacter* strains, it has been shown, that the so-called *pol*-cluster is responsible for pellicle formation [35]. The *polABCDE* cluster shows a high level of homology to the *rfbBACD* genes of Gram-negative bacteria, which are involved in dTDP-rhamnose synthesis [36]. The *polE* gene has already undergone several assignments, since it has a relatively low homology level to glycosyltransferases, in general. A disruption of the *polE* gene in *A. tropicalis* SKU1100. however, leads to a defect in pellicle formation, thereby giving it a central role, either as rhamnosyl-transferase [35], or as galactosyl-transferase [11], which connects the CPS to the cell surface. Moreover, the mutant Pel^−^ cells secreted EPS into the culture medium. To investigate the relationship between *pol*-cluster and pellicle/CPS formation in *K. baliensis* NBRC 16680. *polE* knockouts were carried out in *K. baliensis* NBRC 16680 R, which does not form a *gum*-cluster dependent HePS [20]. Furthermore, we were interested to see, if a *polE* knockout has an effect on the ethanol tolerance of the respective *K. baliensis* strain. The Δ*polE* mutant of *K. baliensis* NBRC 16680 R showed the same rough colony morphology as the rough strain (*K. baliensis* NBRC 16680 R). In contrast to *A. tropicalis* SKU1100. *K. baliensis* NBRC 16680 R Δ*polE* was still able to form a pellicle on the surface, which was, however, only a loose conglomerate of cells and required considerably more time for formation. Furthermore, the Δ*polE* mutant was able to secrete small amounts of EPS into the medium. The incoherent pellicle could therefore be formed by the secreted EPS, thus leading to the formation of a weak EPS/ cell-layer on the surface. The secreted EPS showed a similar composition as the *K. baliensis* NBRC 16680 R capsular HePS. This supports the hypothesis of Ali et al. [11] that PolE is responsible for CPS formation, via addition of some residue(s) that connect the HePS with the cell surface, e.g. β-d-galactopyranosyl residues. A *polE* gene deletion or interruption results in a switch from a rough CPS producing, to an EPS producing phenotype, connected with growth behavior variations [35].

It was shown that a change from a smooth phenotype to the rough phenotype is associated with an increased tolerance against acetic acid [35]. By static cultivation and in combination with growth experiments on NaG plates with different ethanol and acetic acid concentrations of the different *K. baliensis* strains (NBRC 16680, NBRC 16680 R, NBRC 16680 R Δ*polE*), variations in the growth behaviors could be observed. Additionally to *K. baliensis* NBRC 16680 a second *K. baliensis* strain (DSM 14400) was tested, to investigate the variability in the growth behavior between two wild-type strains of *K. baliensis*. The rough mutant strain could form colonies on NaG plates under both ethanol and acetic acid stress in contrast to the wild-type NBRC 16680 even at high acetic acid (0.7%) and ethanol (10%) concentrations. During static growth of *K. baliensis* NBRC 16680 and the rough mutant, a similar pattern compared to the NaG plates appeared, with higher growth rates of *K. baliensis* NBRC 16680 R in NaG medium with acetic acid and ethanol. The Δ*polE* strain, however, showed a diminished growth on acetic acid (up to 0.6%) and ethanol (up to 9%) agar plates, as well as during steady state growth. It is assumed, that the knockout of the *polE* gene results in a change from CPS to EPS, whereas CPS serves as a better barrier against acetic acid [37]. This indicates that the CPS of *K. baliensis* NBRC 16680 R should be involved (inter alia) in the protection against acetic acid and ethanol. The direct relationship between pellicle formation and acetic acid resistance could be proven for *A. pasteurianus* (IFO3283, SKU1108, MSU10), where the rough strains had clearly higher acetic acid resistance abilities than the smooth phenotypes, respectively [19]. The pellicle functions in this case as a biofilm-like barrier and prevents the passive diffusion of acetic acid into the cells [38]. Furthermore, Perumpuli et al. [18] could show, that ethanol in the medium significantly induced pellicle formation. This is in agreement with our observations for *K. baliensis* NBRC 16680, which mutated more frequently in NaG medium with ethanol, suggesting a directed mutation, which could at least partially be triggered under acetic acid fermenting conditions by ethanol or its oxidized product acetic acid.

**Fig. 6:**
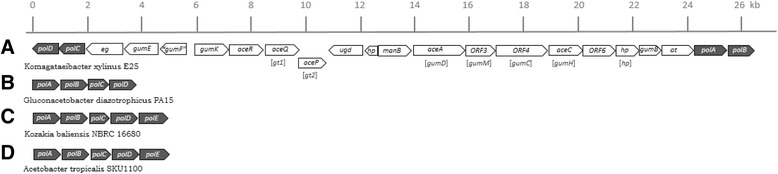
Genetic organization of the *pol*-clusters from different acetic acid bacteria. The *pol*-clusters in combination with the *gum*-like cluster, responsible for the acetan synthesis, of *Ko. xylinus* E25 is depicted in (**a**). The so called acetan cluster has an overall size of ~26 kb and involves 22 genes, including glycosyltransferases (*gt*), mannose-phosphate-guanyltransferase (*mpg*), rhamnosyl transferase (*aceR)*, hypothetical proteins (*hp*), and eight *gum* like genes marked as brackets under the particular genes (*gumB, −C, −D, −E, −F, −H, −K, and –M*). Furthermore, the cluster is flanked with the *polAB* gene upstream and the *polCD* gene downstream of the cluster. The nomenclature for the acetan cluster is based on AM Griffin, Morris, V. J., Gasson, M. J. [33]. In (**b**) is the *pol-*cluster of *Ga. diazotrophicus* Pal5*,* which consists of the *polABCD* genes. **c** and (**d**) show the related *pol*-clusters of *K. baliensis* NBRC 16680 [20] and *A. tropicalis* SKU1100 [35], including the *polABCDE* genes

*K. baliensis* DSM 14400 showed, similar to *K. baliensis* NBRC 16680 R, a high resistance against acetic acid, but was derogated by ethanol concentrations up to 8%. The high tolerance of *K. baliensis* DSM 14400 against acetic acid shows, that this strain has a different defense strategy in dealing with acetic acid, compared to *K. baliensis* NBRC 16680. Both strains were isolated from different environments (DSM 14400 isolated from palm brown sugar, NBRC 16680 isolated from ragi [39]). *K. baliensis* DSM 14400 might, therefore, have been commonly confronted with higher acetic acid concentrations, while they represent a new environmental factor for *K. baliensis* NBRC 16680. In the genome of *K. baliensis* DSM 14400. a cellulose synthase operon was identified on plasmid 3 (pKB14400_3) in contrast to *K. baliensis* NBRC 16680 including genes encoding the three cellulose synthase subunits A, B and C [20], suggesting that additionally formed cellulose could act as barrier against acetic acid. This is also the case for *Komagataeibacter (Ko.) xylinus* E25, isolated from vinegar, which generally deals with high acetic acid concentrations. Via comparison of diverse *pol* clusters from different acetic acid bacteria, a direct genetic connection between the *pol*- and *gum*-like clusters can be observed in *Ko. xylinus* (Fig. 6). *Ko. xylinus* E25 contains no *polE* gene in its genome, but is well known to produce a pellicle polysaccharide consisting of bacterial cellulose [13] and is also able to produce an extracellular HePS called acetan, composed of D-glucose, D-mannose, D-rhamnose, and D-glucuronic acid [6, 40]*.* In case of *Ko. xylinus* E25, the *pol*-cluster is flanking the acetan producing *gum*-like cluster, with the *polAB* gene upstream and the *polCD* gene downstream of the cluster (Fig. 6a). Acetan includes rhamnosyl residues, whose activated form is processed by *polABCD* [35], coding for the enzyme of the TDP-rhamnose synthesis, connecting the acetan synthesis with the enzymes of the *pol*-cluster. The *polE* gene is missing in the *Ko. xylinus* E25 genome (GCA_000550765.1), possibly resulting in no HePS mediated pellicle formation, while a tight cellulose pellicle is commonly formed on the surface of the medium [41], which again contributes to increased acetic acid resistance of the bacterium [42]. This shows that the *gum*-like HePS biosynthesis and the *pol*-cluster are possibly related with each other and are most likely regulated according to the environmental requirements of the respective bacterium. A lack of oxygen, or an increasing ethanol concentration, could be a signal, which leads to an up-regulation of the *polE* gene in *K. baliensis* NBRC 16680. This can take place in association with an ethanol or acetic acid triggered stress response, probably resulting in an induced deactivation of the *gum* clusters, via a transposon insertion.

In the genome of *Gluconacetobacter (Ga.) diazotrophicus* PAl5, no *polE* gene is present as well (Fig. 6b). In contrast to *Komagataeibacter xylinus*, *Ga. diazotrophicus* is an obligate endophytic bacterium that lives symbiotically (N_2_-fixing) in the intercellular space of roots, stem and leaves of sugarcane plants [30]. It produces a HePS composed of D-glucose, D-galactose and D-mannose, whose production is most likely attributable to its *gum*-like cluster similar to *K. baliensis* [31]. The produced HePS plays a key role during plant colonization¸ as shown for the molecular communication between the colonized plants and *Ga. diazotrophicus* [32]. The genetic switch of *K. baliensis* NBRC 16680 wild-type to its rough mutant could indicate an environmentally driven conversion/adaptation of a formerly more plant associated AAB (wild-type) to an increasingly acetic acid producing AAB (rough mutant). Furthermore, *pol*-cluster mediated capsular pellicle polysaccharide biosynthesis and concomitant inactivation of excess HePS production and secretion seems to increase the tolerance against acetic acid in certain AAB such as *K. baliensis* NBRC 16680, while specialized AAB additionally produce tight cellulose pellicles, which are typical for extremely acetic acid tolerant starter cultures used in vinegar production such as *Komagataeibacter xylinus*.

## Conclusion

In summary the results obtained in this study show, that a switch from the *K. baliensis* NBRC 16680 wild-type to a rough mutant strain leads to a significantly increased acetic acid and ethanol resistance. This increased tolerance is probably accompanied by a morphologic switch, from secreted HePS in the wild type to capsular HePS in the rough mutant strain (Fig.  3d). The *polE* gene turned out to be involved for the formation of the resulting CPS. Although the exact role of PolE is still unknown, the results presented here show that *K. baliensis* NBRC 16680 R Δ*polE* displays a reduced tolerance against acetic acid and ethanol, most likely caused by a lack of cell-bound CPS and thus of pellicle formation. Since the morphological change is not reversible, this can also be understood as an adaptive evolutionary step of *K. baliensis* NBRC 16680 resulting in a shift of its ecological niche more towards an acetic acid-rich milieu.

## Additional files

Additional file 1: Primers used for *polE* gene deletion in *K. baliensis* NBRC16680 RTable of primers used fort the *polE* gene deletion in *K. baliensis* NBRC 16680 R. (PPTX 341 kb)

Additional file 2: Deletion of the *polE* gene (A0U90_11950).The deletion mechanism is depicted in (A) displayed with the particular basic vector pKos6b. The deletion of the *polE* gene (A0U90_11950) is shown in (B). In (C) Agarose gel of colony PCR verifying the deletion of the *polE* gene in the genome; lane 1 showing *K. baliensis* NBRC 16680 R with the *polE* gene (3000 bp), lane 2 showing *K. baliensis* NBRC 16680 R with a *polE* deletion (*ΔpolE*), with a PCR product of 1950 bp. (PPTX 418 kb)

## Abbreviations

AA: Acetic acid
AAB: Acetic acid bacteria
*acs*: Cellulose synthesizing operon
*bcs*: Cellulose synthesizing operon
CPS: Capsular polysaccharide
DSM: Deutsche Sammlung von Mikroorganismen
EPS: Exopolysaccharide
EtOH: Ethanol
*G*.: *Gluconobacter*
*Ga.*: *Gluconacetobacter*
Gal: Galactose
Glc: Glucose
*gum*: Protein involved in HePS biosynthesis (derived from *X. campestris*)
HEPES: 2-(4-(2-Hydroxyethyl)-1-piperazinyl)-ethansulfonsäure
HePS: Heteropolysaccharide
HoPS: Homopolysaccharide
HPLC: High-performance liquid chromatography
*K*: *Kozakia*
KM: Kanamycin
*Ko*: *Komagataeibacter*
Man: Mannose
MWCO: Molecular weight cut-off
NaG: Modified sodium-gluconate medium
NBRC: Biological Research Centre NITE, Japan
OD: Optical density
*pol*: Genes involved in pellicle formation
R: Rough strain
*rfb*: Genes involved in dTDP- rhamnose synthesis
*X.*: *Xanthomonas*
