# Supplementary material for: Environmentally triggered genomic plasticity and capsular polysaccharide formation are involved in increased ethanol and acetic acid tolerance in Kozakia baliensis NBRC 16680
Source: BMC Microbiol. 2017 Aug 10;17:172. doi: 10.1186/s12866-017-1070-y (PMC5553594; doi:10.1186/s12866-017-1070-y)
Supplement: Supplementary file 1 — Primers used for polE gene deletion in K. baliensis NBRC16680 RTable of primers used fort the polE gene deletion in K. baliensis NBRC 16680 R. (PPTX 341 kb) [file 12866_2017_1070_MOESM1_ESM.pptx]

## Slide 1
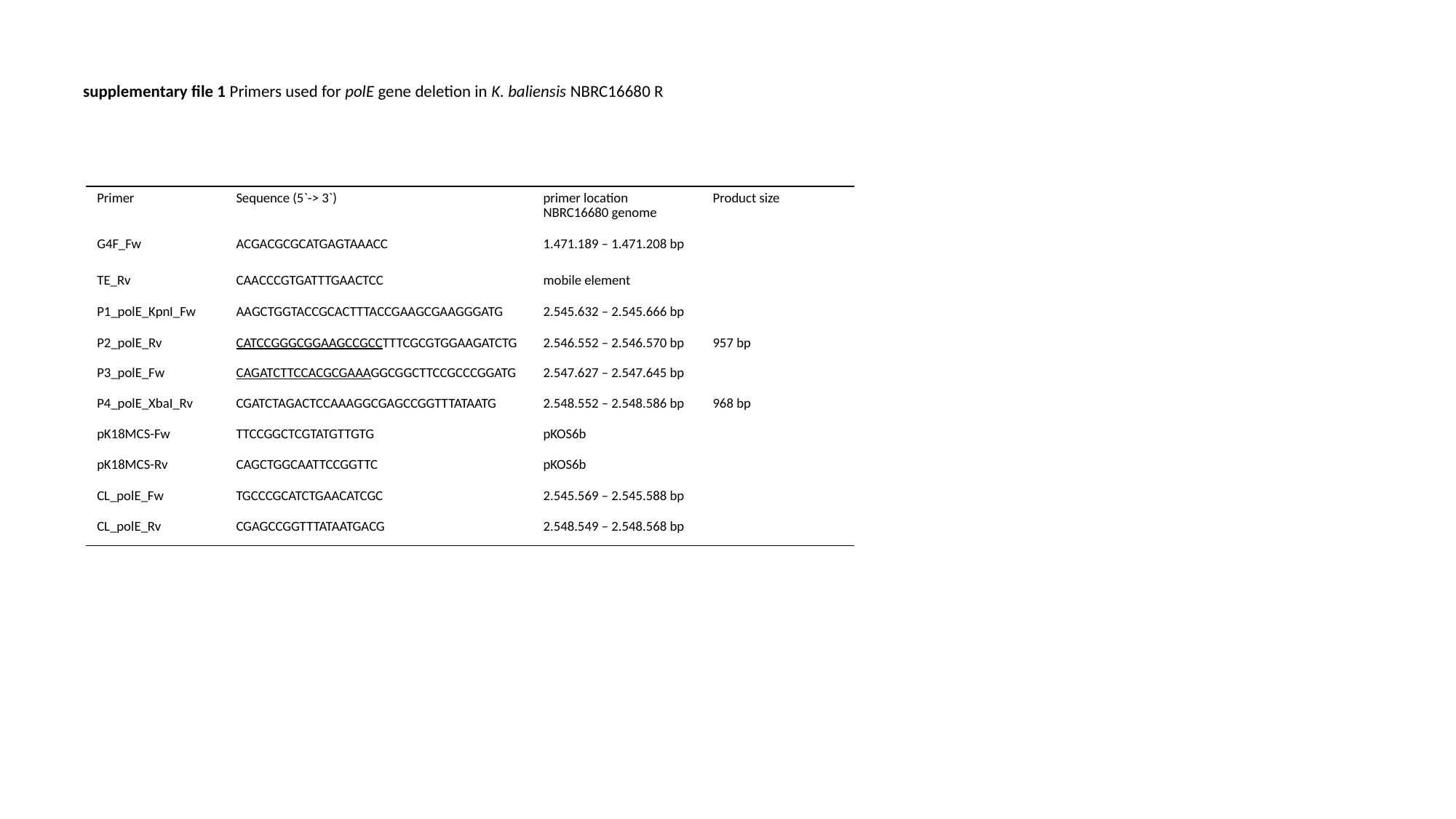

supplementary file 1 Primers used for polE gene deletion in K. baliensis NBRC16680 R
| Primer | Sequence (5`-> 3`) | primer location NBRC16680 genome | Product size |
| --- | --- | --- | --- |
| G4F\_Fw | ACGACGCGCATGAGTAAACC | 1.471.189 – 1.471.208 bp | |
| TE\_Rv | CAACCCGTGATTTGAACTCC | mobile element | |
| P1\_polE\_KpnI\_Fw | AAGCTGGTACCGCACTTTACCGAAGCGAAGGGATG | 2.545.632 – 2.545.666 bp | |
| P2\_polE\_Rv | CATCCGGGCGGAAGCCGCCTTTCGCGTGGAAGATCTG | 2.546.552 – 2.546.570 bp | 957 bp |
| P3\_polE\_Fw | CAGATCTTCCACGCGAAAGGCGGCTTCCGCCCGGATG | 2.547.627 – 2.547.645 bp | |
| P4\_polE\_XbaI\_Rv | CGATCTAGACTCCAAAGGCGAGCCGGTTTATAATG | 2.548.552 – 2.548.586 bp | 968 bp |
| pK18MCS-Fw | TTCCGGCTCGTATGTTGTG | pKOS6b | |
| pK18MCS-Rv | CAGCTGGCAATTCCGGTTC | pKOS6b | |
| CL\_polE\_Fw | TGCCCGCATCTGAACATCGC | 2.545.569 – 2.545.588 bp | |
| CL\_polE\_Rv | CGAGCCGGTTTATAATGACG | 2.548.549 – 2.548.568 bp | |
