# Supplementary material for: Environmentally triggered genomic plasticity and capsular polysaccharide formation are involved in increased ethanol and acetic acid tolerance in Kozakia baliensis NBRC 16680
Source: BMC Microbiol. 2017 Aug 10;17:172. doi: 10.1186/s12866-017-1070-y (PMC5553594; doi:10.1186/s12866-017-1070-y)
Supplement: Supplementary file 2 — Deletion of the polE gene (A0U90_11950).The deletion mechanism is depicted in (A) displayed with the particular basic vector pKos6b. The deletion of the polE gene (A0U90_11950) is shown in (B). In (C) Agarose gel of colony PCR verifying the deletion of the polE gene in the genome; lane 1 showing K. baliensis NBRC 16680 R with the polE gene (3000 bp), lane 2 showing K. baliensis NBRC 16680 R with a polE deletion (ΔpolE), with a PCR product of 1950 bp. (PPTX 418 kb) [file 12866_2017_1070_MOESM2_ESM.pptx]

## Slide 1
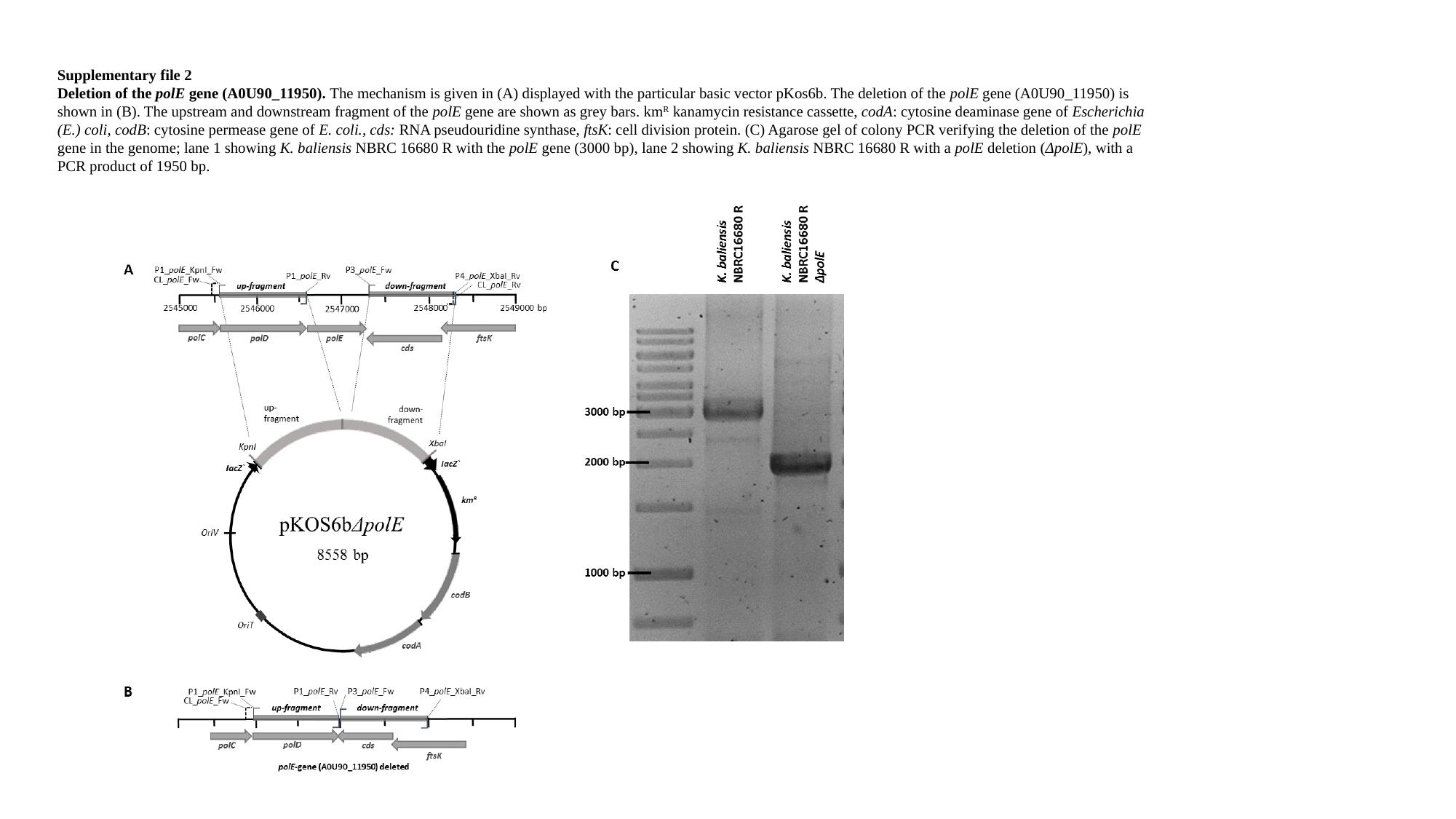

Supplementary file 2
Deletion of the polE gene (A0U90_11950). The mechanism is given in (A) displayed with the particular basic vector pKos6b. The deletion of the polE gene (A0U90_11950) is shown in (B). The upstream and downstream fragment of the polE gene are shown as grey bars. kmR kanamycin resistance cassette, codA: cytosine deaminase gene of Escherichia (E.) coli, codB: cytosine permease gene of E. coli., cds: RNA pseudouridine synthase, ftsK: cell division protein. (C) Agarose gel of colony PCR verifying the deletion of the polE gene in the genome; lane 1 showing K. baliensis NBRC 16680 R with the polE gene (3000 bp), lane 2 showing K. baliensis NBRC 16680 R with a polE deletion (ΔpolE), with a PCR product of 1950 bp.
